# Comparison of 24-Hour urine parameters before and after initiation of Metformin in patients with diabetes and urolithiasis: A retrospective analysis

**DOI:** 10.1007/s00240-025-01881-3

**Published:** 2025-10-27

**Authors:** Taylor Crook, Ian Ong, Yezan Hadidi, Aymon Ali, John M. Hollingsworth, Mary K. Oerline, Vahakn B. Shahinian, Sara Best, Ryan S. Hsi, Joseph J. Crivelli, Ralph V. Clayman

**Affiliations:** 1 Department of Urology, University of California,, Irvine – Orange, CA USA; 2 Department of Urology, University of Michigan, Ann Arbor, MI USA; 3 Department of Urology, University of Florida College of Medicine,, Gainesville, USA; 4 Litholink Corp, IL Itasca, United States of America; 5 Department of Urology, Vanderbilt University Medical Center, Nashville, TN USA; 6 Department of Urology, University of Alabama at Birmingham, AL Birmingham, United States of America

**Keywords:** Urolithiasis, Metformin, Type 2 diabetes, Stone risk

## Abstract

This retrospective study investigated the impact of metformin initiation on 24-hour urine parameters in patients with diabetes with urolithiasis. Utilizing the Medicare-Litholink database, we analyzed 427 patients who started metformin between two 24-hour urine collections conducted less than 18 months apart. To isolate the effect of metformin on urinary parameters as a surrogate marker of stone growth, we excluded patients taking other oral hypoglycemics or medications known to alter urine composition. Our longitudinal analysis revealed no statistically significant changes in key urinary parameters, including supersaturation of calcium oxalate, supersaturation calcium phosphate, and supersaturation of uric acid. Urine volume showed a significant, albeit clinically minor, increase (2.1 to 2.2 L/day, *p* = 0.0074). Time-dependent analysis comparing short-term (< 100 days) and long-term (> 296 days) metformin use also showed no significant impact on urine chemistry. Similarly, when controlling dosage of metformin ($\:\le\:$1000 mg/day or > 1000 mg/day) we found no significant impact on urine chemistry. These findings suggest that metformin use is not significantly associated with reduced urinary stone risk factors in patients with diabetes and urolithiasis, thus, failing to support its use among urolithiasis patients with new onset diabetes.

**Supplementary Information:**

The online version contains supplementary material available at 10.1007/s00240-025-01881-3.

## **Introduction**

Type 2 diabetes mellitus (T2DM) has been well-established as a risk factor for kidney stone formation. Epidemiological studies have demonstrated that patients with diabetes exhibit nearly twice the likelihood of developing urolithiasis compared to non-diabetic individuals [1–4]. This increased susceptibility has been attributed to insulin resistance and associated metabolic alterations, including impaired renal ammoniagenesis, reduced urinary pH, and increased uric acid supersaturation [2]. The mechanistic links between metabolic dysfunction and stone formation have prompted growing interest in exploring the potential impact of oral hypoglycemic medications on modulating stone risk.

Recent studies have explored the effects of oral hypoglycemic drugs such as sodium-glucose cotransporter-2 inhibitors (SGLT-2i), glucagon-like peptide-1 agonists (GLP-1), and dipeptidyl peptidase 4 inhibitors (DPP-4i) on urine chemistry. The main mechanism of action for SGLT-2i is selectively inhibiting sodium-glucose cotransporters in the proximal convoluted tubule, leading to increased urinary glucose excretion and an osmotic diuresis [5]. Several large retrospective cohort studies have reported a significantly lower incidence of urolithiasis in SGLT-2i users compared to patients treated with other hypoglycemics such as GLP-1 agonists or DPP-4i [6–8]. Some of the proposed mechanisms for this benefit are possibly related to an increase in urine volume and citrate excretion along with a reduction in the supersaturation of lithogenic salts [9]– [10].

Metformin, the most widely prescribed oral antihyperglycemic agent in the United States [11], has not been longitudinally investigated in the context of kidney stone prevention in a clinical setting. Its primary mechanism of action involves inhibition of hepatic gluconeogenesis and enhancement of peripheral insulin sensitivity through indirect activation of 5′ adenosine monophosphate-activated protein kinase (AMPK), upregulation of GLUT-4 expression, and increased GLP-1 secretion [12–14]. Since metformin’s introduction and FDA approval in 1994, the tolerability and side effect profile have been found to be safe and are well understood [15]– [16]. Beyond its metabolic benefits, metformin has demonstrated pleiotropic effects in off-label use including reduction in major adverse cardiac events, inhibition of renal fibrosis, decreased end-stage kidney disease, and improved overall survival on unadjusted analysis of patients with renal cell carcinoma [14, 17–21]. Preclinical studies utilizing mouse models have suggested that metformin may also reduce calcium oxalate crystal formation through anti-inflammatory and antioxidant mechanisms; however, the translation of these findings to urolithiasis in the clinical setting has been limited [22–24].

Urine parameters have been widely utilized as surrogate markers to track the effect of drugs on urinary crystal formation, growth and aggregation [25–27]. One study reported that patients with diabetes and urolithiasis exhibited significantly higher total urine volume, citrate, uric acid (UA), sodium, potassium, sulphate, oxalate, chloride, and supersaturation of uric acid (UASS) compared to non-diabetic individuals with urolithiasis [28]. These findings underscore the importance of controlling for diabetic conditions when analyzing the effects of metformin. A recent cross-sectional study by Rosen et al. (2022) [29] failed to demonstrate statistically significant differences in 24-hour urine parameters between patients with diabetes taking metformin and those not using it; however, the single-collection cross-sectional design of this study limited its ability to isolate the specific effects of metformin on urine parameters over time.

Herein is the first longitudinal retrospective cohort study to evaluate the clinical effects of metformin on 24-hour urine parameters in patients with diabetes and urolithiasis. Given that pH, supersaturation of calcium phosphate (CaPSS), supersaturation of uric acid (UASS), and supersaturation of calcium oxalate (CaOxSS) are key surrogate markers of stone risk [26], understanding metformin’s longitudinal impact on urine chemistry offers a valuable opportunity to assess its potential as a stone-preventive agent. From preclinical data, we hypothesized that metformin would favorably alter the urine parameters of patients with diabetes with urolithiasis.

## Methods

Our study leveraged the deidentified Medicare-Litholink database, a comprehensive resource that integrates Medicare claims data from 181,657 beneficiaries with 24-hour urine collection results from patients with diagnosed urolithiasis [30]. This database combines information from multiple Medicare sources (MedPAR, Outpatient, Carrier, and Part D Event Research Identifiable Files) with urine data collected through Labcorp’s Litholink subsidiary from January 1, 2010, to December 31, 2019.

**Fig. 1:**
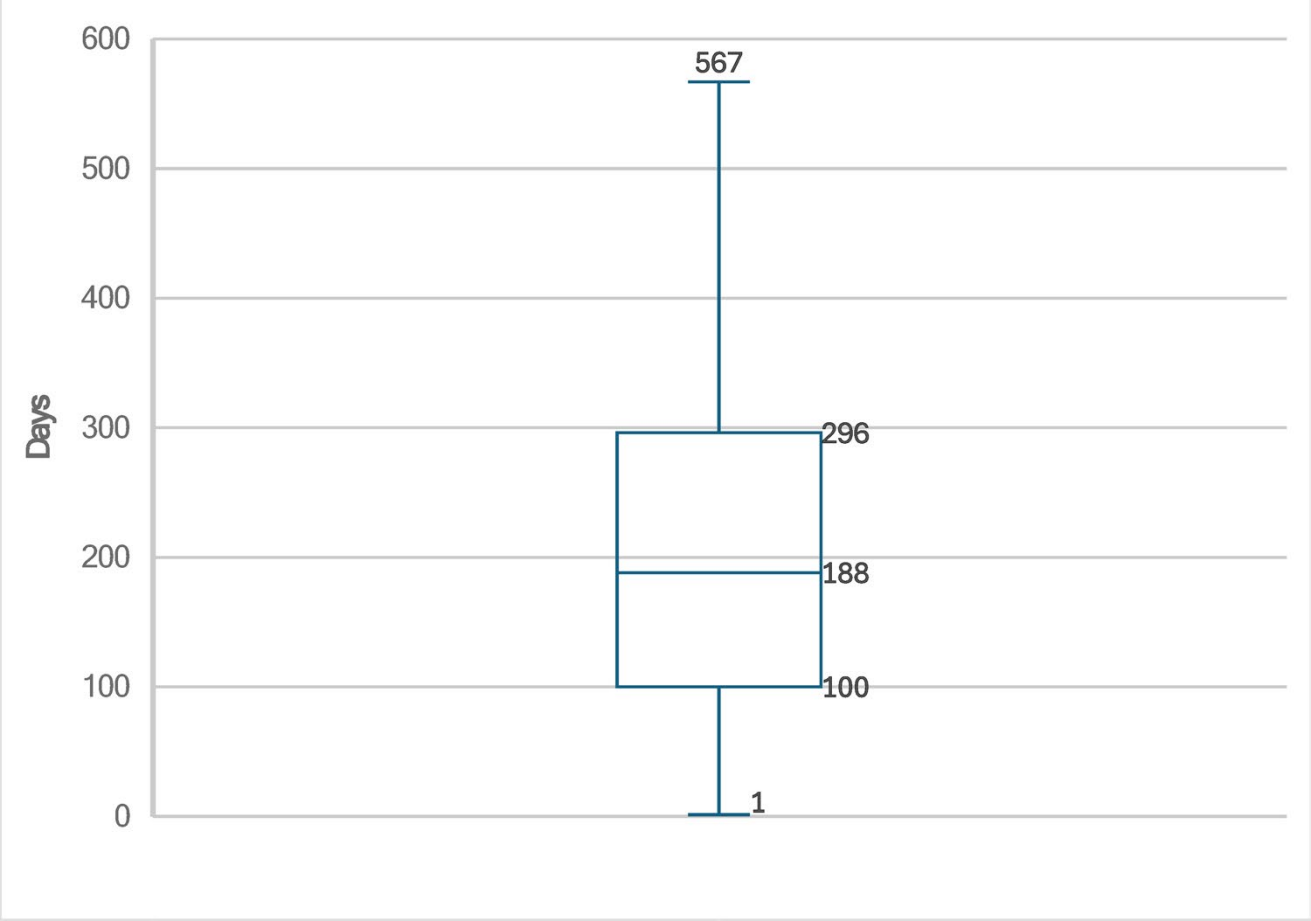
The interquartile ranges for time between the first and second collections

From this dataset, we constructed a longitudinal cohort of urolithiasis diabetic patients (*N* = 427) who had initiated metformin therapy between two 24-hour urine collections conducted less than 18 months apart. Metformin initiation in this study is considered the first prescription fill based on prescription claims data, which is being used as a proxy for medication use. To mitigate potential confounding effects of other medications on urine parameters, we excluded all patients who had been prescribed metformin in the past, other hypoglycemic agents, alkali citrate, or thiazides (Table 1). We characterized and compared the urine chemistry parameters from the first and second 24-hour urine collections. To determine any time-dependent effects on urinary parameters, we stratified this cohort into quartiles based on the duration between collections, focusing our analysis on the first (< 100 days) and fourth quartiles (> 296 days) (Fig. 1). Additionally, we calculated the mean time interval between metformin initiation and the second urine collection.

Our primary outcome was to identify alterations in urine parameters that have been linked with stone growth and formation including CaOxSS, CaPSS, UASS, and pH. Secondary outcomes included the remaining urine parameters that could have an impact on stone risk and growth including oxalate, phosphorus, uric acid, calcium and citrate.

Statistical analysis for the longitudinal comparisons of each first and second collection was performed using a paired t-test with alpha set at 0.05 to allow each patient to act as their own control, eliminating many sources of confounding and isolating the effect of metformin use for each patient. We also performed an independent t-test for the cross-sectional data comparing the Q1 and Q4 urine parameters at the 2nd collection to capture short-term and long-term effects of metformin on urine parameters in similar sized cohorts. To account for multiple comparisons across urinary parameters, we implemented a Bonferroni adjustment which multiplies each p-value by the number of tests that were performed. All analyses were executed using SAS software, Version 9.4 (SAS Institute Inc., Cary, NC).

**Table 1:** Inclusion and exclusion criteria applied to the database of patients with urolithiasis

Inclusion Criteria	Exclusion Criteria
• Patients with two recorded 24-hour urine collections **AND** • Initiation of metformin therapy between the two collections	• If in the interval time between the first and second 24 h urine, they were on potassium citrate or thiazide diuretics **OR** • If the interval time between the first and second 24 h urine is > 18 months **OR** • if patient is < 18 years of age at the time of first collection **OR** • if patient is on any other oral hypoglycemic drug including SGLT-2i, DPP-4i, GLP-1ra, sulfonylureas, thiazolidinediones, and meglitinides

## **Results**

There were 427 patients who met the study’s inclusion criteria. The mean age of these patients was 68 years old (SD, 8.6). The cohort’s racial composition was 89.7% white with 43.8% of the participants being female. The average length of time between the first and second 24 h. urine collection was 206 (SD,139) days (Table 2).

**Table 2:** Demographic and clinical factors of the cohort of patients who initiated Metformin between the two urine collections

Sociodemographic and clinical factors	
Mean age (SD)	68.0 (8.6)
N	427
*Gender %*	
Male	240 (56.2)
Female	187 (43.8)
*Race %*	
White	383 (89.7)
Non-white	44 (10.3)
Mean weight, kg (SD)	92.3 (23.4)
Mean Days Between 1 st and 2nd Collections (SD)	206 (139)
Mean Days Between Initiating Metformin and 2nd Collection (SD)	136 (122)

**Fig. 2:**
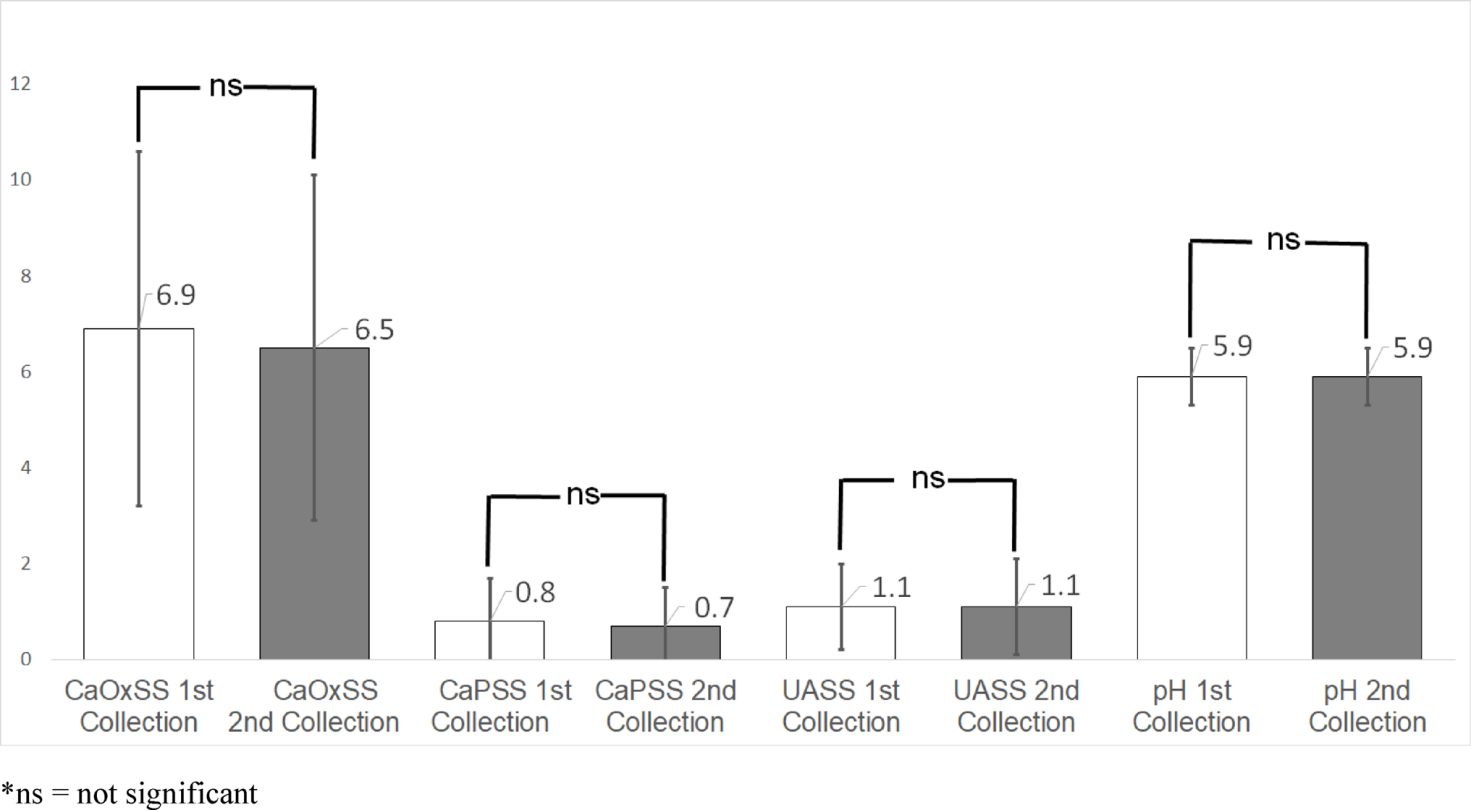
Change between 1 st and 2nd collection for calcium oxalate supersaturation (CaOxSS), calcium phosphate supersaturation (CaPSS), uric acid supersaturation (UASS), and pH. *ns = not significant

According to the study’s primary outcomes, the urine parameters CaOxSS [6.9 versus 6.5; *p* = 1.0000], CaPSS [0.8 versus 0.7; *p* = 0.0946], UASS [1.1 versus 1.1; *p* = 1.0000], and pH [5.9 versus 5.9; *p* = 1.0000] showed no significant change (Fig. 2). Among all urine parameters analyzed, only urine volume showed a statistically significant change between the first and second 24-hour urine collection, increasing from 2.1 L/day to 2.2 L/day (*p* = 0.0074) (Table 3).

**Table 3:** 24-hour urine parameters for first and second collections

Characteristic	Comparison of 1 st and 2nd Collection		
	1 st Collection	2nd Collection	
	(*n* = 427)	(*n* = 427)	*p*-value (Bonferroni adjustment)
Urine factors			
Characteristic	Mean (SD)	Mean (SD)	
pH	5.9 (0.6)	5.9 (0.6)	1.0000
NH4+ (mmol/day)	30.8 (15.0)	31.8 (20.8)	1.0000
Calcium (mg/day)	208.9 (134.0)	203.9 (123.8)	1.0000
Chloride (mmol/day)	180.3 (77.9)	179.3 (74.8)	1.0000
Citrate (mg/day)	715.7 (454.7)	717.7 (436.3)	1.0000
Creatinine (mg/day)	1378.3 (458.2)	1371.7 (442.9)	1.0000
Magnesium (mg/day)	96.9 (48.9)	93.9 (46.7)	1.0000
Oxalate (mg/day)	41.8 (19.3)	41.7 (20.1)	1.0000
Phosphorus (g/day)	0.856 (0.385)	0.816 (0.352)	0.0572
Potassium (mmol/day)	64.8 (25.5)	64.7 (24.3)	1.0000
Sodium (mmol/day)	178.1 (79.8)	174.2 (75.6)	1.0000
Sulfate (mEq/day)	36.4 (18.4)	35.9 (16.8)	1.0000
Urea nitrogen (g/day)	10.7 (4.5)	10.6 (4.1)	1.0000
Uric acid (g/day)	0.622 (0.249)	0.616 (0.228)	1.0000
Calcium Oxalate Supersaturation	6.9 (3.7)	6.5 (3.6)	1.0000
Calcium Phosphate Supersaturation	0.8 (0.9)	0.7 (0.8)	0.0946
Uric Acid Supersaturation	1.1 (0.9)	1.1 (1.0)	1.0000
Net GI alkali absorption	31.5 (26.0)	30.3 (24.5)	1.0000
Urine Volume (L/day)	2.1 (0.8)	2.2 (0.9)	0.0074*

*p < 0.05

We also analyzed the time effect of metformin on the urine parameters at different intervals. The average time from initiating the first prescription fill of metformin to the second urine collection was 136 (SD, 122) days (Table 2). The first quartile (Q1) of patients was defined as patients who had less than 100 days between the first and second 24-hour urine collection and the fourth quartile (Q4) was defined as patients who had more than 296 days between the first and second 24-hour urine collection (Fig. 1). When analyzing the cross-sectional data between Q1 and Q4 patients at their second urine collection, there again were no significant differences in the pre-metformin and post-metformin urine collection chemistries (Table 4). We also looked at the longitudinal data independently for both Q1 and Q4 patients and found no significant change in urinary parameters between the first and second collection for either quartile. For the primary outcomes in Q1, we saw no significant change in CaOxSS [7.1 versus 6.8; *p* = 1.0000], CaPSS [0.8 versus 0.7; *p* = 1.0000], pH [5.9 versys 6.0; *p* = 1.0000] and UASS [1.1 versus 1.1; *p* = 1.0000] (Table 5). Similarly, no significant difference was found among the primary outcomes in the Q4 cohort for CaOxSS [6.1 versus 6.4; *p* = 1.0000], CaPSS [0.8 versus 0.8; *p* = 1.0000], pH [5.9 versus 5.9; *p* = 1.0000] and UASS [1.0 versus 1.0; *p* = 1.0000] (Table 6).

**Table 4:** Urine parameters for the second collection among individuals completing the two tests within the Q1 time interval (< 100 days) and the Q4 time interval (> 296 days)

Characteristic	Cross Sectional Comparison of 2nd Collection Parameters of Q1 and Q4		
	2nd Collection of Q1	2nd Collection of Q4	
	(*n* = 104)	(*n* = 107)	*p*-value (Bonferroni adjustment)
Urine factors			
Characteristic	Mean (SD)	Mean (SD)	
pH	6.0 (0.7)	5.9 (0.6)	1.0000
NH4+ (mmol/day)	31.4 (27.9)	29.6 (15.3)	1.0000
Calcium (mg/day)	187.0 (96.0)	195.3 (112.8)	1.0000
Chloride (mmol/day)	180.0 (76.8)	171.5 (71.9)	1.0000
Citrate (mg/day)	744.5 (427.1)	657.7 (416.3)	1.0000
Creatinine (mg/day)	1300.0 (455.9)	1379.2 (458.9)	1.0000
Magnesium (mg/day)	91.2 (45.5)	95.3 (49.7)	1.0000
Oxalate (mg/day)	44.7 (28.9)	38.1 (13.8)	0.7033
Phosphorus (g/day)	0.769 (0.404)	0.810 (0.334)	1.0000
Potassium (mmol/day)	63.1 (26.0)	62.2 (21.3)	1.0000
Sodium (mmol/day)	176.3 (82.0)	166.0 (74.3)	1.0000
Sulfate (mEq/day)	33.5 (16.9)	34.2 (16.1)	1.0000
Urea nitrogen (g/day)	10.0 (4.0)	10.2 (4.0)	1.0000
Uric acid (g/day)	0.617 (0.243)	0.568 (0.215)	1.0000
Calcium Oxalate Supersaturation	6.8 (3.4)	6.4 (3.5)	1.0000
Calcium Phosphate Supersaturation	0.7 (0.8)	0.8 (0.8)	1.0000
Uric Acid Supersaturation	1.1 (1.0)	1.0 (0.8)	1.0000
Net GI alkali absorption	31.7 (27.3)	27.4 (22.3)	1.0000
Urine volume (L/day)	2.1 (0.9)	2.1 (0.8)	1.0000

**Table 5:** Urine parameters for first and second collections among individuals who completed the two tests within the Q1 time interval (< 100 days)

Characteristic	Days Between Collection < 100 Days (Q1)		
	1 st Collection	2nd Collection	
	(*n* = 104)	(*n* = 104)	*p*-value (Bonferroni adjustment)
Urine factors			
Characteristic	Mean (SD)	Mean (SD)	
pH	5.9 (0.6)	6.0 (0.7)	1.0000
NH4+ (mmol/day)	28.0 (13.5)	31.4 (27.9)	1.0000
Calcium (mg/day)	202.6 (133.7)	187.0 (96.0)	1.0000
Chloride (mmol/day)	180.1 (79.1)	180.0 (76.8)	1.0000
Citrate (mg/day)	766.9 (479.5)	744.5 (427.1)	1.0000
Creatinine (mg/day)	1334.0 (515.4)	1300.0 (455.9)	1.0000
Magnesium (mg/day)	97.5 (56.7)	91.2 (45.5)	0.8160
Oxalate (mg/day)	43.9 (27.7)	44.7 (28.9)	1.0000
Phosphorus (g/day)	0.811 (0.429)	0.769 (0.404)	1.0000
Potassium (mmol/day)	66.9 (30.3)	63.1 (26.0)	0.9852
Sodium (mmol/day)	176.2 (79.9)	176.3 (82.0)	1.0000
Sulfate (mEq/day)	35.8 (20.2)	33.5 (16.9)	1.0000
Urea nitrogen (g/day)	10.4 (4.8)	10.0 (4.0)	1.0000
Uric acid (g/day)	0.627 (0.261)	0.617 (0.243)	1.0000
Calcium Oxalate Supersaturation	7.1 (3.5)	6.8 (3.4)	1.0000
Calcium Phosphate Supersaturation	0.8 (0.9)	0.7 (0.8)	1.0000
Uric Acid Supersaturation	1.1 (0.9)	1.1 (1.0)	1.0000
Net GI alkali absorption	34.2 (25.9)	31.7 (27.3)	1.0000
Urine volume (L/day)	1.9 (0.7)	2.1 (0.9)	1.0000

**Table 6:** Urine parameters for first and second collections among individuals who completed the two tests within the Q4 time interval (> 297 days)

Characteristic	Days Between Collection > 297 Days (Q4)		
	1 st Collection	2nd Collection	
	(*n* = 107)	(*n* = 107)	*p*-value (Bonferroni adjustment)
Urine factors			
Characterstic	Mean (SD)	Mean (SD)	
pH	5.9 (0.5)	5.9 (0.6)	1.0000
NH4+ (mmol/day)	29.8 (14.2)	29.6 (15.3)	1.0000
Calcium (mg/day)	195.3 (116.7)	195.3 (112.8)	1.0000
Chloride (mmol/day)	170.3 (60.9)	171.5 (71.9)	1.0000
Citrate (mg/day)	619.1 (377.6)	657.7 (416.3)	1.0000
Creatinine (mg/day)	1357.8 (417.7)	1379.2 (458.9)	1.0000
Magnesium (mg/day)	95.4 (45.3)	95.3 (49.7)	1.0000
Oxalate (mg/day)	39.2 (15.1)	38.1 (13.8)	1.0000
Phosphorus (g/day)	0.850 (0.339)	0.810 (0.334)	1.0000
Potassium (mmol/day)	62.2 (20.9)	62.2 (21.3)	1.0000
Sodium (mmol/day)	169.2 (65.7)	166.0 (74.3)	1.0000
Sulfate (mEq/day)	36.3 (17.4)	34.2 (16.1)	1.0000
Urea nitrogen (g/day)	10.6 (4.0)	10.2 (4.0)	1.0000
Uric acid (g/day)	0.577 (0.204)	0.568 (0.215)	1.0000
Calcium Oxalate Supersaturation	6.1 (3.3)	6.4 (3.5)	1.0000
Calcium Phosphate Supersaturation	0.8 (0.9)	0.8 (0.8)	1.0000
Uric Acid Supersaturation	1.0 (0.8)	1.0 (0.8)	1.0000
Net GI alkali absorption	29.4 (25.4)	27.4 (22.3)	1.0000
Urine volume (L/day)	2.0 (0.8)	2.1 (0.8)	1.0000

When controlling dosage effects of metformin on the urine parameters, we found no statistical significance between patients who took $\:\le\:$1000 mg/day versus >1000 mg/day. The cohort who was prescribed $\:\le\:$1000 mg/day had no significant change in CaOxSS [7.1 versus 6.6; *p* = 0.7494], CaPSS [0.9 versus 0.8; *p* = 0.7458], pH [5.9 versus 5.9; *p* = 1.0000] and UASS [1.1 versus 1.0; *p* = 1.0000] (Table 7). The cohort prescribed >1000 mg/day had no significant change in CaOxSS [6.4 versus 6.3; *p* = 1.0000], CaPSS [0.7 versus 0.6; *p* = 0.7343], pH [5.9 versus 5.9; *p* = 1.0000] and UASS [1.1 versus 1.1; *p* = 1.0000] (Table 8).

**Table 7:** Urine parameters for first and second collections among individuals who were prescribed $\:\le\:$1000 mg/day of Metformin

Characteristic	Patients Prescribed $\:\le\:$1000 mg/day of Metformin		
	1 st Collection	2nd Collection	
	(*n* = 286)	(*n* = 286)	*p*-value (Bonferroni adjustment)
Urine factors			
Characterstic	Mean (SD)	Mean (SD)	
pH	5.9 (0.6)	5.9 (0.6)	1.0000
NH4+ (mmol/day)	30.7 (14.8)	31.6 (19.7)	1.0000
Calcium (mg/day)	217.1 (134.6)	208.4 (116.4)	1.0000
Chloride (mmol/day)	175.2 (79.1)	173.2 (73.5)	1.0000
Citrate (mg/day)	697.4 (430.2)	705.6 (413.0)	1.0000
Creatinine (mg/day)	1360.5 (433.1)	1358.0 (434.0)	1.0000
Magnesium (mg/day)	97.9 (48.1)	93.8 (47.1)	1.0000
Oxalate (mg/day)	39.6 (14.0)	39.7 (16.6)	1.0000
Phosphorus (g/day)	0.879 (0.370)	0.840 (0.344)	0.3565
Potassium (mmol/day)	63.8 (25.0)	63.3 (23.6)	1.0000
Sodium (mmol/day)	174.2 (80.3)	169.9 (74.9)	1.0000
Sulfate (mEq/day)	35.7 (17.9)	35.8 (17.3)	1.0000
Urea nitrogen (g/day)	10.5 (4.4)	10.5 (4.2)	1.0000
Uric acid (g/day)	0.606 (0.233)	0.605 (0.224)	1.0000
Calcium Oxalate Supersaturation	7.1 (3.8)	6.6 (3.5)	0.7494
Calcium Phosphate Supersaturation	0.9 (0.9)	0.8 (0.8)	0.7458
Uric Acid Supersaturation	1.1 (0.9)	1.0 (0.9)	1.0000
Net GI alkali absorption	30.7 (24.5)	29.5 (22.7)	1.0000
Urine volume (L/day)	2.0 (0.9)	2.2 (0.8)	0.1502

**Table 8:** Urine parameters for first and second collections among individuals who were prescribed > 1000 mg/day of Metformin

Characteristic	Patients Prescribed > 1000 mg/day of Metformin		
	1 st Collection	2nd Collection	
	(*n* = 141)	(*n* = 141)	*p*-value (Bonferroni adjustment)
Urine factors			
Characterstic	Mean (SD)	Mean (SD)	
pH	5.9 (0.6)	5.9 (0.7)	1.0000
NH4+ (mmol/day)	30.8 (15.6)	32.2 (23.1)	1.0000
Calcium (mg/day)	192.1 (132.3)	194.8 (137.0)	1.0000
Chloride (mmol/day)	190.4 (74.7)	191.6 (76.4)	1.0000
Citrate (mg/day)	752.9 (500.1)	742.2 (480.8)	1.0000
Creatinine (mg/day)	1414.4 (505.0)	1399.3 (460.7)	1.0000
Magnesium (mg/day)	94.9 (50.5)	94.2 (46.1)	1.0000
Oxalate (mg/day)	46.2 (26.7)	45.6 (25.3)	1.0000
Phosphorus (g/day)	0.811 (0.413)	0.769 (0.364)	1.0000
Potassium (mmol/day)	66.9 (26.3)	67.7 (25.5)	1.0000
Sodium (mmol/day)	186.0 (78.3)	182.7 (76.5)	1.0000
Sulfate (mEq/day)	37.7 (19.6)	36.0 (15.7)	1.0000
Urea nitrogen (g/day)	11.2 (4.7)	10.8 (3.7)	1.0000
Uric acid (g/day)	0.654 (0.278)	0.639 (0.234)	1.0000
Calcium Oxalate Supersaturation	6.4 (3.5)	6.3 (3.8)	1.0000
Calcium Phosphate Supersaturation	0.7 (0.9)	0.6 (0.8)	0.7343
Uric Acid Supersaturation	1.1 (0.9)	1.1 (1.0)	1.0000
Net GI alkali absorption	32.9 (28.9)	31.7 (28.0)	1.0000
Urine volume (L/day)	2.1 (0.8)	2.3 (0.9)	0.3241

## **Discussion**

In the diabetic population at risk for urolithiasis, hypoglycemic drugs along with GLP-1 agonists have been studied as a potential pharmacologic preventative therapy. SGLT-2i have been associated with the reduction of stone incidence risk compared to most other hypoglycemic drugs [6]. Notably lacking in this analysis, until the present study, has been a definitive review of metformin, the most widely prescribed oral antihyperglycemic agent. There has been one earlier study of the impact of metformin on urine chemistry in the diabetic urolithiasis population; however, this was a cross-sectional study with a small cohort of patients with diabetes taking metformin as monotherapy, which precluded the study from isolating the effect of metformin on urine chemistry over the time prior to and then after initiating metformin therapy [29].

Preclinical molecular studies have postulated that metformin may attenuate stone risk through the downregulation of key inflammatory modulators, notably monocyte chemoattractant protein 1 (MCP-1) and osteopontin (OPN) [22]– [23]. Similarly, ex-vivo studies showed that metformin inhibited CaOx stone formation and destabilized already formed monohydrate stones however the mechanism of action was not clearly discerned [24]. Based on these mechanistic insights, we hypothesized that metformin initiation could lead to favorable alterations in urinary parameters associated with increased stone risk, particularly CaOxSS, UASS, pH and CaPSS.

Our longitudinal analysis of patients with diabetes with urolithiasis who initiated metformin between 24-hour urine collections revealed that metformin was not meaningfully associated with changes in their urine parameters. Although an increase in urine output was significant, the increase from 2.1 L to 2.2 L is too small to be clinically relevant and could be attributed to lifestyle changes noted as the *stone clinic effect* whereby patients are encouraged to increase their fluid intake; the standard goal for urine output and decreased stone risk is 2.5 L [31]. Indeed, the baseline urine output for these patients was already high, leaving little room for a clinically meaningful increase in urine output attributable to metformin. Furthermore, the duration of metformin use, whether less than 100 days or more than 296 days, did not impact urine chemistry demonstrating that there was not a time dependent impact on urine chemistry.

This discrepancy between benchtop and animal findings versus clinical outcomes underscores the complexity of translating molecular mechanisms into observable physiological changes in human subjects and highlights the multifactorial nature of human kidney stone formation. Our findings corroborate and expand the sole earlier cross-sectional study which reported no significant differences in urinary parameters between patients with diabetes taking metformin and those not taking the medication [29]. While that study provided insights into the lack of observable differences in a single collection, it did not provide information regarding the temporal effects of metformin on urinary parameters. Our longitudinal approach addresses this limitation by tracking changes over time, offering a more comprehensive analysis of metformin’s impact on urine chemistry by using each patient as their own control. To further attempt to isolate any specific effects of metformin on stone forming urinary diathesis, we implemented rigorous exclusion criteria, eliminating patients using other hypoglycemic agents or medications known to affect urine parameters, such as thiazides or citrate enhancing substances. The present study is further strengthened due to the utilization of clinical data from a large sample; this approach increased both the robustness and generalizability of our results. Nonetheless, there may be an impact of metformin on calcium oxalate stone disease that lies outside the realm of the current metabolic evaluation of a urolithiasis patient. Certainly, an explanation of the earlier cited findings of metformin inhibition of CaOx stone formation and destabilization of already formed monohydrate stones requires further study [24].

Several limitations of our study warrant consideration. First, we note that filling the prescription is not akin to actually taking the medication; clearly, it is possible that some proportion of our patient cohort either did not take any of the Metformin they received from their pharmacy or failed to follow the prescription instructions and thus did not take the full daily dose. Also, we did not control for variation in patient diets either, which could have further confounded our results; however, the analysis of urine tests within the same individual mitigates this concern to some degree. Furthermore, our study did not stratify patients based on stone composition due to the unavailability of this information. Given the heterogeneity of kidney stone types, particularly the distinction between calcium oxalate (CaOx) and other stone compositions, this lack of stratification may have obscured potential subgroup effects of metformin on specific stone types (e.g., uric acid, brushite, struvite, or cystine). It is also important to acknowledge the limitations of using urinary parameters as surrogate markers for stone risk. While these parameters are widely accepted and associated with stone risk, more objective measures of stone risk, such as imaging studies or long-term follow-up of stone events, would provide a more detailed analysis of metformin’s impact on stone formation.

## Conclusion

Metformin remains a first-line treatment for type 2 diabetes due to its well-established glycemic, cardiovascular, and chronic kidney disease benefits. Despite preclinical evidence suggesting metformin might ameliorate stone burden, our analysis reveals no clinically significant changes in key urinary risk factors for stone formation following metformin initiation. This suggests that metformin should not be prescribed as first line therapy for stone prevention in diabetic urolithiasis patients, especially in light of the benefit in this dual disease patient population of the SGLT-2i drugs. On the other hand, metformin had no adverse effect on urinary stone risk and thus could reasonably be combined with other hypoglycemics to control glycemic levels when necessary.

## Supplementary Information

Below is the link to the electronic supplementary material.

Supplementary Material 1

## Data Availability

Data is provided within the manuscript and supplementary information files.
